# A New Validation Methodology for In Silico Tools Based on X-ray Computed Tomography Images of Tablets and a Performance Analysis of One Tool

**DOI:** 10.3390/pharmaceutics13091488

**Published:** 2021-09-16

**Authors:** Sebastian Bollmann, Peter Kleinebudde

**Affiliations:** Institute of Pharmaceutics and Biopharmaceutics, Heinrich Heine University Duesseldorf, Universitaetsstrasse 1, 40225 Duesseldorf, Germany; sebastian.bollmann@hhu.de

**Keywords:** X-microCT, in silico development, validation, formulation development, dissolution testing, dissolution prediction

## Abstract

In silico tools which predict the dissolution of pharmaceutical dosage forms using virtual matrices can be validated with virtual matrices based on X-ray micro-computed tomography images of real pharmaceutical formulations. Final processed images of 3 different tablet batches were used to check the performance of the in silico tool F-CAD. The goal of this work was to prove the performance of the software by comparing the predicted dissolution profiles to the experimental ones and to check the feasibility and application of the validation concept for in silico tools. Both virtual matrices based on X-ray micro-computed tomography images and designed by the software itself were used. The resulting dissolution curves were compared regarding their similarity to the experimental curve. The kinetics were analysed with the Higuchi and Korsmeyers–Peppas plot. The whole validation concept as such was feasible and worked well. It was possible to identify prediction errors of the software F-CAD and issues with the virtual tablets designed within the software.

## 1. Introduction

In 2004, the American Food and Drug Administration (FDA) started the Process Analytical Technology initiative to transform the art of pharmaceutical development and processes to science with the goal of quality by design [1]. Since then the concept was revised several times. The International Council of Harmonisation adopted the concept for several guidelines [2,3]. Since the recent years, an increasing number of in silico tools to support the pharmaceutical development is available. However, a complete in silico development of pharmaceutical dosage forms does not seem possible at the present time. Nevertheless, in silico tools may contribute to cost reduction and saving of development time in the future.

Nowadays, different software packages like F-CAD (CINCAP, Basel, Switzerland) or DDDPlus (Simulations Plus Inc., Lancaster, CA, USA) provide assistance with the formulation development. The different in silico software tools are either based on cellular automata or the discrete element method and finite element method, respectively [4,5]. The example software F-CAD of this work used cellular automata and differential equations [6]. The concept of cellular automata was described by Wolfram (2002) and can be used to calculate complex correlations or phenomena with simple rule sets [7].

Virtual matrices were used as pharmaceutical dosage forms for the in silico calculations. The software can either design virtual dosage forms with a defined composition or upload Tagged Image File Format (TIFF) images as virtual matrix [6,8,9,10]. Kimura et al. calculated the disintegration time of mefenamic acid containing tablets with a sufficient accuracy using F-CAD in 2013 [8]. The second goal of F-CAD is the prediction of dissolution profiles [6].

A reliable validation method for in silico tools is a mandatory step to enable a complete in silico development without experimental fitting for all pharmaceutical applications. The correctness of the prediction has to be verified. The opportunity of uploading TIFF images as virtual matrix for the in silico simulation provides the possibility to compare the predicted dissolution profile of X-ray micro-computed tomography (XμCT) images of dosage forms with the experimental dissolution profile of the imaged dosage forms. If both profiles match, the in silico tool can predict the behaviour of the API and the excipient(s) correctly. Furthermore, the virtual matrices designed by the software could be compared to the virtual matrices based on XμCT images to verify the correct virtual design of the tool.

If the prediction of a formulation is correct, the data can possibly be used to build up databases of excipients or placebo mixtures to transfer the prediction to new drugs, assuming negligible interactions between the different compounds. The potential was shown by Bollmann and Kleinebudde in a proof of concept study [11]. Due to limitations of the XμCT imaging contrasts between pharmaceutical powders, complex formulations could be accessible under the condition of negligible interactions between all excipients and using multiple binary mixtures for validation purposes. This would allow a complete in silico formulation development in line with the concept of quality by design [10,11]. However, the potential and the feasibility of this methodology needs to be further investigated.

The in silico tools require specific properties of XμCT images to upload them as virtual matrix. The image processing of the XμCT images has to guarantee that the resulting virtual matrix matches the real tablet. The pathway from the raw image to the uploadable TIFF image of tablets was presented for the tool F-CAD by Bollmann and Kleinebudde [9,10]. Some of the resulting images were used as virtual matrices for the prediction of dissolution profiles in this work [10]. The predicted dissolution profiles of the virtual matrices either based on the XμCT images or designed by the particle arrangement and compaction module (PAC-module) of F-CAD were compared to the experimental dissolution profiles.

Costa and Lobo reviewed several approaches to compare dissolution profiles [12]. In this work, the similarity ($f_{1}$ and $f_{2}$ factor) of the predicted profiles compared to the experimental one is calculated as it is the recommended approach by the FDA and European Medicines Agency [12,13,14]. Furthermore, the kinetics are analysed with the Higuchi and Korsmeyer–Peppas plot to determine the release mechanism [15,16,17,18].

The goal of this work is to verify and rate the prediction of dissolution profiles of the software package F-CAD and to prove the concept and feasibility of the validation of in silico tools using XμCT images as virtual matrices. In addition, a sensitivity analysis with respect to virtual matrices based on XμCT images with different desirabilities is performed to prove the hypothesis of Bollmann and Kleinebudde about the impact of the desirability on the in silico dissolution calculation.

## 2. Materials and Methods

### 2.1. Excipients and Tableting

Theophylline anhydrate (BASF, Ludwigshafen, Germany, Dx50 114 μm) served as model active pharmaceutical ingredients (API) while ethyl cellulose (Aqualon N10, Ashland, Wilmington, DE, USA, Dx50 210 μm) was used as matrix former. Binary mixtures (100 g) in different ratios (25/75, 50/50, and 75/25) were blended with a Turbula mixer TC2 (Willy A. Bachofen AG, Muttenz, Switzerland) with 49 rpm for 15 min. Round, flat-faced tablets were compressed with a compaction pressure of approximately 160 MPa on a Styl’One Evolution (MEDEL’Pharm, Beynost, France). The target weight was 400 mg with a diameter of 11.28 mm. The tablets were already used in previous publications [9,10]. The data were used for additional analysis methods in this paper. The batches were named TxxEyy and the tablets were numbered. T25E75-01 means the tablet 1 of the batch containing 25 % theophylline and 75 % ethyl cellulose.

### 2.2. Characterisation

The helium density of both compounds was determined at 25 °C using an AccuPyc 1330 (Micromeritics, Unterschleißheim, Germany (*n* = 3)). The tablets were characterised in height, diameter, and weight using a SmartTest50 (Sotax AG, Basel, Switzerland). More details are available in section 2.5 of the previous publication [9].

The solubility of the API was determined UV-spectroscopically (UV-1800 SHIMADZU, Kyoto, Japan). A suspension of theophylline in phosphate buffer pH 6.6 of the United States Pharmacopeia (USP) was stored and stirred in a water bath (37 °C) for three weeks (*n* = 3). The suspension was filtered (0.2 μm, polypropylene), diluted and measured at a wavelength of 271 nm.

### 2.3. Dissolution Testing

Dissolution tests were performed with an automatic dissolution tester (DT 720, ERWEKA, Langen, Germany) using apparatus 2 (USP) and 1000 mL phosphate buffer pH 6.6 (USP). The stirring speed was set at 100 rpm and the temperature was kept constant at 37 °C. Samples were taken every ten minutes through frits (Poroplast, 10 μm). The drug release was determined UV-spectroscopically (UV-1800 SHIMADZU, Kyoto, Japan) in flow-through cuvettes at a wavelength of 271 nm. The total API content was determined by the plateau after complete dissolution of the tablets [9].

### 2.4. Imaging and Image Processing

The tablets were imaged by a CT-ALPHA (ProCon X-ray, Sarstedt, Germany). The amperage was 100 μA and the voltage 86 kV. In total, 1600 projections were taken while the sample rotated 360° with a voxel size of 7 μm.

The reconstructed raw images (VGStudio 3.0.1, Volume Graphics GmbH, Heidelberg, Germany) were processed and prepared for the software package F-CAD (Special edition with 28 μm voxel size instead of 50 μm, TD Version: 2.8.1.14, FCalc Version = 1.0.5.55652, CINCAP, Basel, Switzerland) with the open source software ImageJ (version 1.52p) [19]. The software package F-CAD consists of three different modules: tablet designer module (TD-module), PAC-module and dissolution simulation module (DS-module). The details of the image processing are available in the previous publications [9,10]. The simulations were performed with two GEFORCE RTX 2080Ti grafic cards, 128 GB RAM and CPU (Intel®Xeon®E5-2630 v3).

#### Nomenclature and Methods of Pathways

The following notations were used for the different image processing pathways: bleach correction: reference (Ref) [20], stack (Sta) [21,22]; lighting correction: remove background (RB) [23], pseudo flat field correction (PFF) [24], uncorrected (UC); filtering: non-local means (NLM) [25,26], uncorrected (UC); contrast adjustment: gamma (G) [27], enhance contrast (EC) [28], uncorrected (UC); segmentation: multilevel thresholding: k-means clustering (cluster number) (KM(X)) [29,30], binary thresholding: default iterative intermeans (D) [31], Huang’s fuzzy 2 (H) [32] (implemented by Schindelin), intermodes (IM) [33], isodata (ID) [31], Li’s minimum cross entropy (L) [34,35,36], maximum entropy (MaE) [37], mean (M) [38], minimum error (MiE) [39], minimum (Mi) [33], moments (Mo) [40], Otsu’s threshold clustering (O) [41], percentile (P) [42], Renyi’s entropy (R) [37], triangle (T) [43], Yen’s thresholding (Y) [36,44], ignore black voxel: iB, ignore white voxel: iW [45]. The source code of the binary thresholdings can be found in [45]. The different processing methods were written in the applied order from the left to the right and separated by “/”. The combination of two binary thresholdings were indicated by “//”. The image was copied twice before the segmentation process. One binary thresholding method was applied to each copy and both obtained thresholds were used to segmentate the original image. For example, the description Ref/RB/UC/G3.7/EC18/D-iBiW//R-iB means that the pathway bleach corrected by a reference image, lighting corrected with a low degree polynomial function, no filtering, gamma function with the input value 3.7, contrast enhanced with 18 % saturated voxels and thresholds determined with Default’s threshold algorithm ignoring black and white voxels and Renyi’s entropy algorithm ignoring black voxels. The details of the entire image processing evaluation and the different methods can be found in the previous publications [9,10].

### 2.5. Matrices of the In Silico Dissolution Calculation

Table 1 depicts the different matrices of batch T25E75 which were used for the in silico dissolution calculation. The corresponding matrices of the batches T50E50 and T75E25 can be found in the Appendix A (Table A1 and Table A2).

A sensitivity analysis was performed on the the third tablet of each batch. Bollmann and Kleinebudde (2021a,b) evaluated the image processing pathways for four binary mixtures (2 APIs, 2 excipients) in three different ratios each (12 batches). All pathways were analysed for different levels: API specific (all tablet images of batches containing the same API), combination specific (all tablet images of batches containing the same API and excipient), batch specific (all tablet images of one batch—same API, excipient and their ratio), and tablet specific. The pathways were ranked according to their desirability. The desirability was determined for the recovery rates of API mass, excipient mass and total mass and their respective ratios. The tablet desirability was calculated as geometric mean value of these six desirability values, each characterising a parameter of the virtual matrix. The API, combination or batch desirability, respectively, was calculated as the geometric mean of the geometric mean tablet desirabilities and a corresponding precision factor. A desirability of 1 is the optimum of the specified parameters. For more details on desirability assessment and image processing evaluation, see Bollmann and Kleinebudde (2021a,b) [9,10]. The final processed images of the best two pathways of the API specific (API1 and API2) and combination specific image processing (C1 and C2) were applied, as well as ones of the four highest performing batch specific image processings (B1–B4). Additionally, pathways with tablet specific desirabilities from 0.1 to 1 (increment 0.1) were used to analyse the sensitivity of the desirability (T1–T11).

Images of two batch specific pathways of the first and the fifth tablet were used to investigate the precision of the calculation of images of one batch. Furthermore, virtual tablets were designed by the TD-module and produced by the PAC-module to compare the dissolution profiles of the virtual tablets with the processed images of real tablets. The virtual tablets were produced in two different ways. API and excipient were either distributed randomly within the matrix (D) or random seeds were distributed and grown until the target tablet mass was obtained (G). Four different virtual tablets were designed for batch T25E75, both two with distributed API and excipient (D, D2) and two with seeded and grown compounds (G, G2).

### 2.6. Parameters of the In Silico Dissolution Calculation

Table 2 depicts the input parameters of the dissolution calculation. The voxel edge length was 28 μm like in the images. The number of voxels in each dimension was set to the actual image dimensions. The dissolution profiles were predicted for 12 h with samplings every ten minutes. The mixing parameter was used to take the convection of the paddle stirrer into account. The identification numbers of the compounds were set to the actual grey values of the compounds in the image stack. The density of the phosphate buffer was determined by weighting a tarred volumetric flask. The molar mass of the compounds was taken from the certificates of analysis. The contact angle of ethyl cellulose was determined using a drop shape analyser (DSA 100, Krüss GmbH, Hamburg, Germany). The pore size was estimated after consultation with the developer of the F-CAD software package. The viscosity [46] and tension [47] of the phosphate buffer were taken from the literature.

The tablet porosity was calculated with Equation (1)

(1) $$\begin{array}{c} \phi_{tablet}=\left(1-\frac{m_{total}^{2}}{\pi \times {\left(0.5\times D\right)}^{2}\times h\times \left(m_{API}\times \rho_{API}+m_{ex}\times \rho_{ex}\right)}\right)\times 100\% \end{array}$$

with $\phi_{tablet}$ as the tablet porosity, $m_{total}$ as total mass of the tablet, *D* as diameter of the tablet, *h* as tablet height, $m_{API}$ as API mass, $\rho_{API}$ as density of the API, $m_{ex}$ as excipient mass, and $\rho_{ex}$ as density of the excipient. It was used to determine the virtual porosity of the excipient for the dissolution simulation calculated by Equation (2)

(2) $$\begin{array}{c} \phi_{ex}=\frac{S\times \left(\phi_{tablet}-\phi_{Image}\right)\times Vx_{size}\times \left(Vx_{API}+Vx_{ex}\right)\times \left(1+0.01\times \phi_{tablet}\right)}{h\times Vx_{ex}} \end{array}$$

with $\phi_{ex}$ as assumed excipient porosity for the dissolution calculation, *S* as number of slices of the image stack, $\phi_{tablet}$ as tablet porosity, $\phi_{Image}$ as porosity of the virtual tablet in the image, $Vx_{size}$ as edge length of a voxel, $Vx_{API}$ as number of voxels of the API, $Vx_{ex}$ as number of voxels of the excipient, and *h* as tablet height. The porosity of the virtual tablet in the images was calculated by the PAC-module.

### 2.7. Analysis of the Dissolution Profiles

#### 2.7.1. Difference and Similarity Factors

The dissolution profiles were statistically analysed using python 3.8 [48]. The first parameter was the $f_{1}$ factor calculated using Equation (3)

(3) $$\begin{array}{c} f_{1}=\frac{\sum_{t=1}^{n}\left|R_{t}-T_{t}\right|}{\sum_{t=1}^{n}R_{t}}\times 100 \end{array}$$

with $f_{1}$ as the difference factor of the calculated dissolution profiles, $R_{t}$ as cumulative percentage dissolved of the reference and, $T_{t}$ as cumulative percentage dissolved of the tested profile. The $f_{2}$ values were obtained by applying Equation (4)

(4) $$\begin{array}{c} f_{2}=50\times log\left(\frac{100}{\sqrt{1+\frac{1}{n}\sum_{t=1}^{n}{\left(R_{t}-T_{t}\right)}^{2}}}\right) \end{array}$$

with $f_{2}$ as the similarity factor of the calculated dissolution profiles, $R_{t}$ as cumulative percentage dissolved of the reference and, $T_{t}$ as cumulative percentage dissolved of the tested profile. The calculated dissolution profiles were compared to the experimental determined dissolution profile [49]. For the calculation, either all data points of the first 12 h of the experimental dissolution testing or all data points up to the complete drug release (if < 12 h) and the corresponding ones of the predicted profiles were used.

#### 2.7.2. Kinetic Analysis

The profiles were analysed regarding their kinetics by using the Higuchi and Korsmeyers–Peppas plot [15,16,17,18]. The linear regression line was calculated for all data points within 20% to 60% drug release. If less then 4 data points were in this range, the profile was excluded from the kinetic analysis. The equations of the linear regression lines and the adjusted $R^{2}$ of both plots were used to determine the kinetics. Additionally, slope and intercept of the regression line of the simulated dissolution profile were statistically tested against the regression line of the experimental dissolution profile using *t*-tests [50,51].

## 3. Results and Discussion

### 3.1. Determined Parameters

The results of the compound characterisation to determine the parameters for the dissolution simulation can be found in Table 2. The helium density of theophylline and ethyl cellulose was already determined for a previous publication [9]. The contact angle was determined as 93.2 ± 1.0°. The DS-module assumed that the tablets could not be wetted because the contact angle was above 90°. However, it was known from the experiments that the tablets were wetted. The DS-module only used the contact angle of the individual compounds, but not the one of the mixture. This may led to the wrong assumption. Therefore, the contact angle of ethyl cellulose was set to 89° which was the closest value to the determined parameter and led to wettability in the prediction.

### 3.2. Computation Performance

Although the dissolution testing lasted up to 72 h, the prediction of the dissolution profiles was only performed for the first 12 h due to the calculation time (approximately 13 h calculation time for 12 h dissolution time when running two simulations simultaneously, each using one GEFORCE RTX 2080Ti grafic card). The experimental dissolution testing is usually performed with six individual tablets. Therefore, the experiment was approximately three times faster than the in silico prediction of the DS-module. The calculation speed is dependant on the number of CUDA cores, the processor speed, and the speed of the memory. Therefore, it would be possible to reduce the calculation time with different hardware. In addition to the stand-alone version used in this study, there will be a cloud version available soon. This should accelerate the calculation as well. The deviations of the predicted profiles from the experimental profile after 12 h were estimated from the predicted profiles and the calculated parameters of the plots.

### 3.3. Batch T25E75

**Figure 1:**
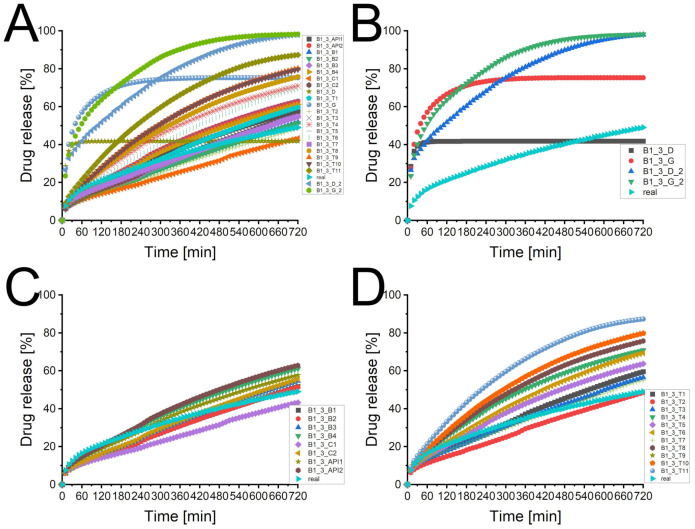
The predicted dissolution profiles of different image processing pathways of the imaged tablet 3 of batch T25E75 (*n* = 1). The experimental determined dissolution profile is depicted as well. (**A**) Overview of all dissolution profiles of the imaged tablet 3 of batch T25E75. CAVE: only the symbol and colour of the experimental curve remains always the same. (**B**) Comparison of experimental dissolution profile and both types of virtual tablets produced by the PAC-module. (**C**) Comparison of experimental dissolution profile and virtual tablets based on XμCT images (API-, combination-, and batch specific pathways). (**D**) Comparison of experimental dissolution profile and virtual tablets based on XμCT images (tablet specific pathways) with different desirabilities.

Figure 1A depicts the experimental determined dissolution curve and different predicted dissolution profiles. There was a broad scattering of the predicted profiles using processed XμCT images as matrix with a tendency to higher deviations with a lower desirability of the matrix. The two dissolution profiles of the virtual produced tablets (T25E75-03_D and T25E75-03_ G) differed considerably from the experimental one and the XμCT image based predicted profiles. Figure 1B depicts that the virtual tablets produced by the PAC-module had a low performance. Both were considerably faster dissolving and reached a plateau at about 42% or 75% of released API after one or three hours, respectively. This indicates that the API was not percolating the virtual matrices. The experimental dissolution reached a plateau after approximately 72 h (data not shown). The API was released completely. Therefore, the API was percolating the tablet. The XμCT image based matrices seem to represent the percolating API structure. However, the kinetics of the dissolution profiles seem to be different from the experimental one. The graphs displayed in Figure 1C did not match the experimental profile but they were close to it. There was no clear increase in the performance visible for a higher specification of the image processing (API vs. combination vs. batch specific). This was surprising because a more specific image processing should increase the desirability. The sensitivity analysis Figure 1D depicts the broad scattering of the predicted profiles described above. Although the tendency was not clearly observed for the different specification levels, there was a dependency of desirability and prediction performance. A higher desirability led to a predicted profile closer to the experimental one. However, there were exceptions like T25E75-03_ T7. Therefore, there has to be a factor for the image quality which was not full described by the desirability. In spite of high performance of the API specific pathways, a combination specific image processing is still recommended because it increases the probability of higher desirabilities. Therefore, the recommendations of Bollmann and Kleinebudde [10] are still valid.

Table 3 displays the statistical analysis of the predicted profiles compared to the experimental one. Most $f_{2}$-values of the XμCT image based predicted profiles were above 50 and most $f_{1}$-values were lower than 15 which is the originally used limit for the similarity of dissolution profiles [49]. The most common used limit is a $f_{2}$ factor above 50 [12,13,14] which was obtained by even more virtual matrices. Although there were some exceptions (e.g., T7), the $f_{1}$ values were higher and the $f_{2}$ values lower for lower desirabilities. This confirmed the observed tendency.

**Figure 2:**
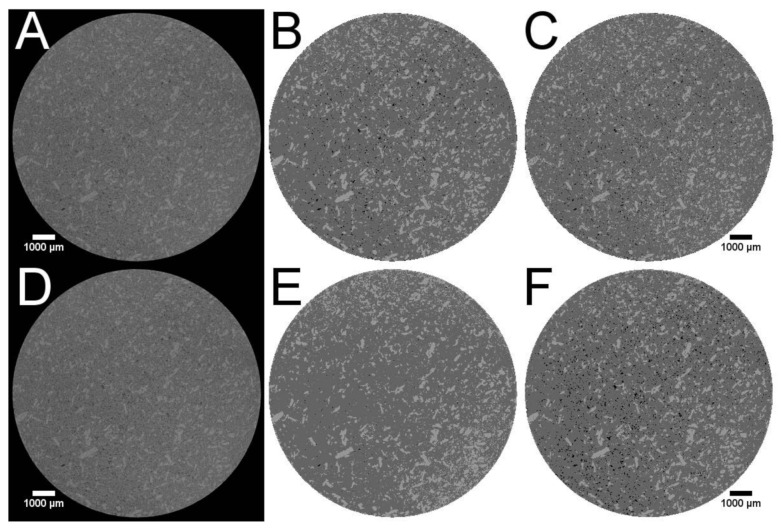
Cross-section 84 of different virtual tablets based on the XμCT image of tablet 3 of batch T25E75. Theophylline is always displayed as light grey, ethyl cellulose as dark grey, and air as black. The scale of all images is equal. (**A**,**D**) Binned XμCT image. (**B**) Virtual tablet T1. (**C**) Virtual tablet T2. (**E**) Virtual tablet T4. (**F**) Virtual tablet T7.

Figure 2 illustrates the background of these exceptions. The $f_{1}$ and $f_{2}$ value of the virtual tablets T1 (Figure 2B) and T2 (Figure 2C) were similar. The pattern matched both well to the raw image (Figure 2A). The slight differences of T1 and T2 were caused by a higher amount of assumed small particles (T2 > T1). The dissolution curve of T1 matched better to the beginning of the experimental curve, T2 was superior for the last part of the profile. Although the virtual tablet T7 had only a desirability of 0.506, the $f_{1}$ and $f_{2}$ values were superior. The pattern of the API was close to the one of T1. Therefore, the dissolution profile of the matrix matched the first part of the experimental curve. The recovery rates were 84.3% (API) and 99.9% (excipient) [10]. Some parts of the API particles were misallocated as excipient, some parts of the excipient particles were assumed as air. The overestimated pore size accelerated the calculated drug release. Therefore, most parts of the calculated profile matched the experimental curve. However, it is obvious, that a longer calculation time would increase the deviation because the difference of the profiles increased with the time. The cross-section of T4 explains the low performance of a matrix with a comparable high desirability (Figure 2E, 0.801). The recovery rates were 110.5% and 98.3% [10]. The overestimation of API removed almost all pores and led to artificial fines at the edges of the matrix. The DS-module corrected the lower porosity, as described in Section 2.6. The artificial fines led to a faster drug release and a considerable difference to the experimental curve. This emphasises the crucial importance of a visual verification of the image processing. The desirability increases the probability of a suitable virtual matrix but does not guarantee it. It was only one matrix used for each desirability except for the desirability of 1. If all matrices would be used to calculate the dissolution profile, it would likely confirm the hypothesis, that most matrices with a higher desirability are superior. However, this needs to be investigated. This may provide information how the desirability calculation could be improved to reduce the user dependency of the visual verification.

**Figure 3:**
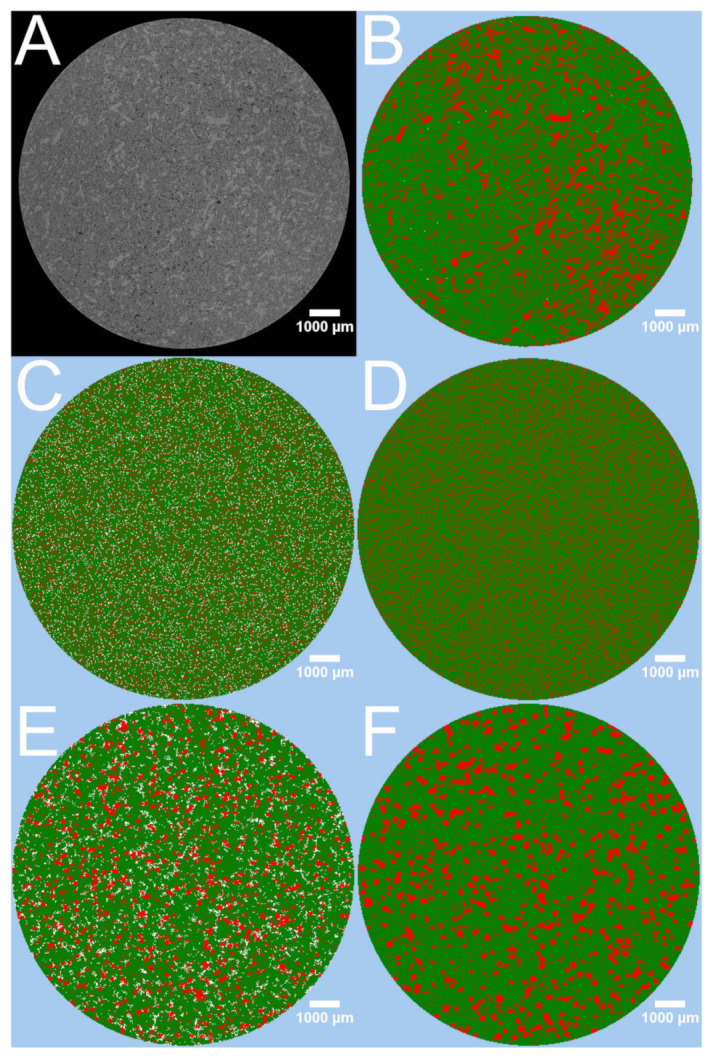
Different cross-sections of tablet 3 of batch T25E75. Theophylline is represented as light grey or red, ethylcellulose as dark grey or green, and air as black or white, respectively. (**A**) Cross-section 88 of the binned XμCT image. (**B**) Cross-section 88 of the virtual tablet created by the PAC-module using the final processed XμCT image of the pathway B1. (**C**) Cross-section of the virtual tablet obtained by distributing the target masses of the compounds. (**D**) Cross-section of the virtual tablet obtained by distributing the both compounds in the determined ratio until the interparticular porosity is 0. (**E**) Cross-section of the virtual tablet obtained by distributing seeds and growing them to the target masses of the compounds. (**F**) Cross-section of the virtual tablet obtained by distributing seeds and growing them in the determined ratio until the interparticular porosity is 0.

The profiles of the virtual tablets produced by the PAC-module were inferior. The $f_{1}$ and $f_{2}$ values of the virtual tablet using the distributed compounds were superior to the one using the grown compounds which was caused by the lower plateau. If the prediction was performed over a longer time period, the results would shift. Figure 3 depicts the differences of the virtual tablets created by the PAC-module and the image of the tablet. The XμCT based matrix (Figure 3B) had a comparable distribution of API and excipient as the binned raw image (Figure 3A). The virtual matrix produced with the PAC-module distributing the compounds (Figure 3C) had a completely different appearance, while the virtual matrix obtained by seeding and growing (Figure 3E) appeared similar. Both virtual matrices of the PAC-module had the overestimation of the pore size in common. Because both matrices reflected the full porosity of the tablet, there was no internal correction by the DS-module. Therefore, all API particles which were included by the excipient ethyl cellulose were not released. The fast increase in released drug and the plateau of the dissolution profiles were the product of too large assumed pores and a non-percolating API pattern of the virtual tablets. The particle size was designed by clustered voxels but it was not possible to check the particle size distribution and number of particles within the virtual tablets in the PAC-module. The seeded and grown virtual tablet was built by visual perception. The design of the seeded and grown virtual tablets was user depended because the user decided the seeded mass of each compound (determines the number of particles) and the number of growing steps (determines the particle size). Therefore, the designed virtual tablets may were not the optimum.

Artificial virtual tablets produced by the PAC-module, which kept the ratio of API and excipient constant but reduced the interparticular porosity to 0 (Figure 3D: distributing compounds, Figure 3F: seeding and growing compounds), changed the calculated dissolution profiles. Figure 1B depicts the differences. The internal correction of the porosity (the difference of the determined porosity and the interparticular porosity of the virtual tablet is assumed as intraparticular porosity) was able to mimic a percolating API cluster and the drug release was slowed down. Nevertheless, both profiles were inferior to the XμCT based virtual matrices and the calculated drug release was considerably faster than the experimental one. The matrix with the distributed compounds was superior to the seeded and grown one. This was caused by the small particle size and the higher number of particles.

The Higuchi plot of the experimental dissolution profile indicates, as expected, a square root of time kinetic. The Korsmeyers–Peppas plot confirm the assumption as the parameter n was 0.48 which is close to the 0.45 proposed for a cylinder and Fickian diffusion as the transport mechanism of the API [12]. The slopes of both all XμCT image based matrices and the virtual tablets produced by the PAC-module were significantly different from the experimental determined drug release profile. The XμCT image based matrices obtained values for the parameter n in the range of 0.60 to 0.75 in the Korsmeyers–Peppas plot, while the parameter n was always lower than 0.4 for the virtually produced tablets by the PAC-module. The DS-module predicted an anomalous transport of the API for XμCT based virtual matrices [12]. Although the similarity of many XμCT image based matrices was given in accordance to the guidance of the FDA and EMEA [13,14], the profile itself differs significantly. Costa and Lobo pointed out some weaknesses of the $f_{1}$ and $f_{2}$ factors which were confirmed by the results presented [12]. Both kinetics and similarity did not match for the virtual tablets of the PAC-module. It is not recommendable to use the similarity of dissolution profiles to validate in silico predicted dissolution profiles. The kinetics and mechanisms of the drug release should be analysed as well.

**Figure 4:**
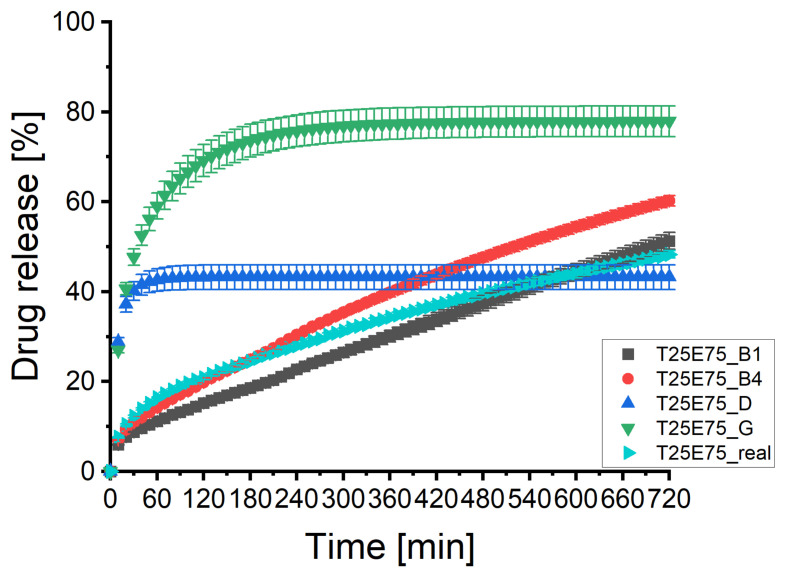
The predicted dissolution profiles of two image processing pathways of the imaged tablets 1, 3, and 5 of batch T25E75 ($\bar{x}\pm SD$, *n* = 3). The experimental determined dissolution profile is depicted as well.

Figure 4 depicts the mean dissolution profiles of three tablets. The standard deviations of the experimental mean dissolution profile were low. The two mean curves of the XμCT image based matrices obtained similar standard deviations. The standard deviation of the virtually produced tablets were slightly increased but acceptable as well. The shape of the profiles did not change. Therefore, the calculations of the DS-module were reproducible and reflected the experimental deviations of different tablets. The different shapes of the predicted dissolution profiles seem to be systematic and should be investigated in more detail to improve the software package. Table A3 of the Appendix A depicts the data of the statistical analysis of the profiles. The results were in line with the results described for tablet 3. All slopes were highly significant different from the experimental curve.

### 3.4. Batch T50E50

**Figure 5:**
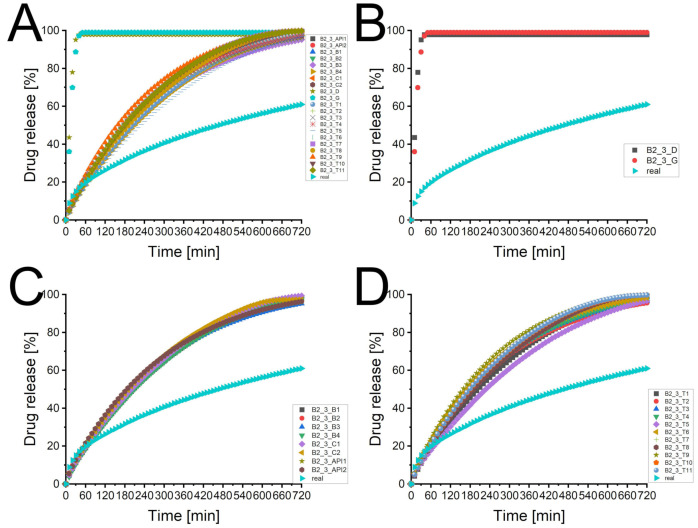
The predicted dissolution profiles of different image processing pathways of the imaged tablet 3 of batch T50E50 (*n* = 1). The experimental determined dissolution profile is depicted as well. (**A**) Overview of all dissolution profiles of the imaged tablet 3 of batch T50E50. CAVE: only the symbol and colour of the experimental curve remains always the same. (**B**) Comparison of experimental dissolution profile and both types of virtual tablets produced by the PAC-module. (**C**) Comparison of experimental dissolution profile and virtual tablets based on XμCT images (API-, combination-, and batch specific pathways). (**D**) Comparison of experimental dissolution profile and virtual tablets based on XμCT images (tablet specific pathways).

Figure 5A illustrates the predicted and experimental determined dissolution profiles of the third tablet of batch T50E50. The experimental dissolution profile reflects a slowly eroding ethyl cellulose matrix. The profiles of the XμCT image based matrices were much less scattering compared to the ones described in Section 3.3, but the drug release was in all cases considerably faster than determined by experiment. The decreased scattering reduced the importance of the desirability and the visual verification since the sensitivity of the DS-module for those differences was lower. The virtual tablets of the PAC-module had a percolating API cluster but the drug release was completed within 40 min in both cases (Figure 5B). This was probably caused by the assumption of artificial large pores, as shown in Figure 3. There was no clear difference between the matrices based on the API, combination or batch specific image processing (Figure 5C). Although all curves showed a considerably slower dissolution than the virtual tablets produced by PAC-module, the predicted dissolution profiles were considerably faster than the experimental one. There seem to be a slight tendency, but the sensitivity analysis indicates only a negligible impact of the desirability on the prediction (Figure 5D). This supports the conclusion of Section 3.3. The desirability represented not all important parameters and the combination specific image processing is still recommended as compromise between effort and outcome.

The results of the statistical analysis are presented in Table A4 of the Appendix A. The $f_{1}$ and $f_{2}$ factors confirmed a dissimilarity for all predicted profiles and the differences of the slopes of the Higuchi and Korsmeyers–Peppas plot were always highly significant. The parameter n of the Korsmeyers–Peppas plot was 0.47 for the experimental profile which is close to the 0.45 proposed for a cylinder and Fickian diffusion as the transport mechanism of the API as well [12]. Therefore, the erosion of the matrix was slower than the drug release. The parameter n was between 0.7 and 0.8 for all virtual matrices based on XμCT images. The DS-module predicted again an anomalous transport of the API for XμCT based virtual matrices [12] but the results were closer to the zero order kinetic than to the Fickian diffusion. This indicates that the DS-module overestimated the erosion of the matrices. It was not possible to calculate the kinetics of the virtual tablets produced by PAC-module due to the fast predicted drug release. The performance of the DS-module was worse compared to Section 3.3. The systematic error needs to be investigated to get reliable predictions of dissolution profiles.

**Figure 6:**
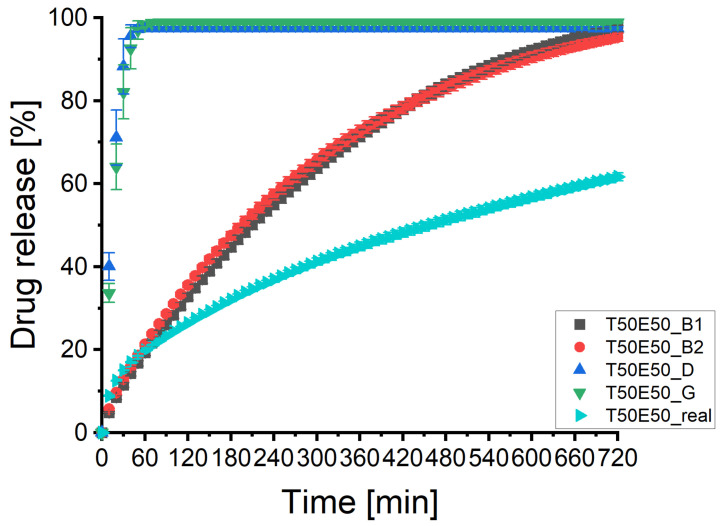
The predicted dissolution profiles of two image processing pathways of the imaged tablets 1, 3, and 5 of batch T50E50 ($\bar{x}\pm SD$, *n* = 3). The experimental determined dissolution profile is depicted as well.

Figure 6 illustrates the mean dissolution profiles of three tablets. As described in Section 3.3, the shapes of the profiles remained identically with small standard deviations in all cases. Table A3 of the Appendix A depicts the data of the statistical analysis of the profiles. The results supported the results described for tablet 3. All profiles were dissimilar and the slopes were highly significant different from the experimental curve. This supports the assumption of a systematic calculation error within the software package which has to be investigated and corrected.

### 3.5. Batch T75E25

**Figure 7:**
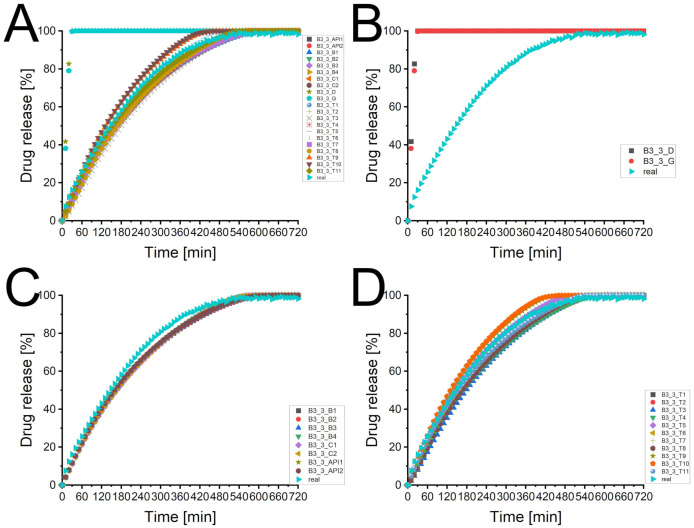
The predicted dissolution profiles of different image processing pathways of the imaged tablet 3 of batch T75E25 (*n* = 1). The experimental determined dissolution profile is depicted as well. (**A**) Overview of all dissolution profiles of the imaged tablet 3 of batch T75E25. CAVE: only the symbol and colour of the experimental curve remains always the same. (**B**) Comparison of experimental dissolution profile and both types of virtual tablets produced by the PAC-module. (**C**) Comparison of experimental dissolution profile and virtual tablets based on XμCT images (API-, combination-, and batch specific pathways). (**D**) Comparison of experimental dissolution profile and virtual tablets based on XμCT images (tablet specific pathways).

Figure 7A displays the predicted and experimental determined dissolution profiles of tablet 3 of batch T75E25. The different virtual matrices based on XμCT images resulted in only small differences of the predicted dissolution profiles. The profiles matched the experimental curve well. The profiles of the virtual produced matrices by the PAC-module were considerably faster releasing the drug and different from the other profiles. Figure 7B illustrates the considerable difference of the virtual tablets designed by the PAC-module. Figure 7C depicts that there was no relevant difference between virtual matrices based on different specific XμCT image processing pathways. Figure 7D displays the sensitivity analysis. There was no clear pattern visible. This underlines the conclusions drawn in Section 3.4. The desirability described not all relevant parameters. The recommendation to use the combination specific pathways stands because the API specific image processing led to huge variabilities within the image processing.

Table A5 of the Appendix A depicts the results of the statistical analysis of the profiles. The virtual produced tablets by the PAC-module were dissimilar to the experimental curve and a kinetic analysis was not possible due to the fast drug release. This was probably caused by the assumption of artificial large pores, as shown in Figure 3. All virtual matrices based on XμCT images were similar to the experimental profile. The $f_{1}$-value was always lower than 9.5 and the $f_{2}$-value always above 58. They all passed the originally determined limits ($f_{1}$< 15 and $f_{2}$> 50) [49] and fulfil the requirements of the FDA and EMEA [12,13,14]. There seem to be no dependency of the similarity and the desirability left because there was no trend visible. This would reduce the effort of the image processing and the visual verification considerably. The parameter n of the Korsmeyer–Peppas plot was 0.74 for the experimental curve and indicates anomalous transport of the API [12]. The matrix was eroding. Therefore, the kinetic depended on the erosion and Fickian diffusion. The parameter n of all predicted matrices were statistically significant different from the experimental curve but the *p*-value increased considerably compared to Section 3.3 and Section 3.4 (from range 10${}^{-59}$ to 10${}^{-113}$ to range 10${}^{-4}$ to 10${}^{-13}$). The slopes of the Higuchi plot mostly were not statistically significant different from the one of the experimental profile but the intercepts were always statistically significant different.

**Figure 8:**
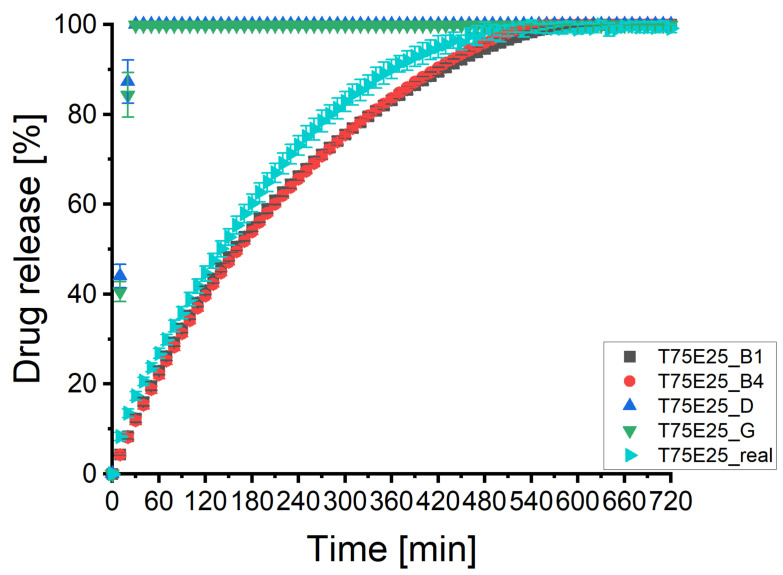
The predicted dissolution profiles of two image processing pathways of the imaged tablets 1, 3, and 5 of batch T75E25 ($\bar{x}\pm SD$, *n* = 3). The experimental determined dissolution profile is depicted as well.

Figure 8 illustrates the mean dissolution profiles of three tablets. The shapes of the profiles remained the same by low standard deviations. Therefore, the results were reproducible. The results of the statistical analysis (Table A3 in the Appendix A) were in line with the results presented above. The DS-module was able to predict the dissolution profiles of batch T75E25. In contrast to the results of Section 3.3 and Section 3.4 the statistical significant deviations had no practical relevance if virtual matrices based on XμCT images were used.

The sensitivity of the DS-module regarding the virtual matrices based on XμCT images decreased with increasing API content of the tablets. This indicates more effort and a critical image processing, as well as visual verification for lower API doses. The reason for this phenomenon may be the reduced impact of the excipient on the dissolution profile. The drug release of the fast eroding tablets (batch T75E25) was predicted well by the DS-module. This indicates that the dissolution of the API was predicted correctly. However, the erosion of the matrix was probably overestimated for batch T50E50. This resulted in a too fast predicted drug release. If the Fickian diffusion through a matrix was the drug releasing mechanism, the scattering of the drug releasing profiles of different virtual matrices increased. Therefore, the image processing seems to be more crucial for matrix based systems. The significant different kinetics of the predicted curves and the good prediction of batch T75E25 suggest a systematic error of the Fickian diffusion calculation of the API. The predicted transport mechanisms were different (Fickian diffusion vs. anomalous transport). This needs to be investigated and fixed.

The virtual tablets produced by the PAC-module behaved considerably different from the virtual matrices based on XμCT images and always were inferior. The virtual tablets of the PAC-module did not reflect the structure of the real tablets and led to considerably wrong profiles. Therefore, the design of those virtual tablets has to be revised critically and has to be changed until real tablets can be represented correctly. It may be possible to build similar tablets with the used PAC-module version with high effort but the benefits of the in silico tool would be lost. Although the software performed well for the eroding batch, it has to be improved to be a useful tool for the dissolution prediction in general.

With the current software version, the prediction performance is expected to be high for virtual matrices based on XμCT images of tablets which dissolution is determined by the solubility of the API (fast eroding systems, immediately release, low solubility of the API or large crystals). The data were reported to CINCAP so that future versions of F-CAD might be able to predict other systems, such as (slow eroding) matrices correctly with virtual matrices based on both XμCT images and the software itself.

## 4. Conclusions

The virtual tablets produced by the the PAC-module of F-CAD did not represent the real tablets. The predicted dissolution profiles were significantly different from the experimental profiles and the XμCT image based predicted profiles. It is mandatory to critically revise the design of virtual tablets within the software. In spite of a sufficient prediction of the dissolution of eroding matrices (batch T75E25) using XμCT image based matrices, the DS-module of software was not able to predict the dissolution of tablets with Fickian diffusion as drug releasing mechanism (batches T25E75 and T50E50) correctly. A systematic error in the calculation of these systems was observed which needs to be investigated. It is not recommended to judge the performance of in silico tools predicting dissolution profiles only by the $f_{1}$ and $f_{2}$ similarity [49] but also by the release mechanism and the respective kinetics to avoid misinterpretations (depicted with batch T25E75). The presented work emphasises the need of external validation methods of in silico tools. If an in silico tool designs virtual dosage forms and the software parameters are fitted until the predicted curve matches the experimental one, an optimisation within a certain design space is possible but no complete in silico development will be achievable. Additionally, the software may seem to be more accurate than it really is because some potential prediction errors could be concealed by over-fitting. The parameters have all to be determinable by the individual compounds and the in silico tools have to calculate the resulting physical-chemical properties of the mixture correctly. Otherwise, a complete in silico formulation development will not be possible. This is still a long way to go. Virtual matrices based on XμCT images provide the possibility to represent the real structure and distribution of pharmaceutical dosage forms. They can be used to validate both the virtually produced dosage forms of in silico tools and their performance. They provide the possibility to identify systematic errors and to optimise the performance of in silico tools. If the predicted dissolution curve considerably deviates from the experimental profile of the imaged dosage form, the known calculations of the tool may serve as starting point to investigate the differences. Furthermore, the use of virtual matrices based on XμCT images in in silico tools may help to increase the understanding of different mechanisms and interactions. XμCT image based matrices were successfully used to rate the performance of an in silico tool. The validation methodology presented was feasible to distinguish between a reliable and poor performance and to detect prediction errors of an in silico tool. The whole concept and work flow from the imaging and image processing [9,10] to the upload and calculation of virtual matrices based on XμCT images with an in silico tool worked well. However, there are still some improvements mandatory to use the presented methodology as a validation method. The sensitivity of the tested software package F-CAD regarding the desirability of the processed XμCT images decreased with an increasing API content. Therefore, both the image processing and visual verification becomes more critical with lower drug loads. One possible reason for this phenomenon could be the calculation issue of the software. Another important reason could be the pattern of the API and excipient within the virtual tablet. Although the overall pattern was similar, there were considerable differences regarding the particle number and size. The pattern was rated by visual perception and not included in the calculation of the desirability. However, the pattern seems to be an important parameter because the performance of the virtual matrices was not completely linked to the desirability. Therefore, either the visual verification has to be done carefully for all matrices with a high desirability (leads to a strong user dependency) or the pattern has somehow to be included in the desirability calculation. More investigations of this phenomenon are mandatory to understand the issue and to optimise the methodology.

## Figures and Tables

**Table 1:** The selected images for the in silico calculations. The line called real displays the experimentally determined data of the real tablet.

Name	Tablet	Pathway	Tablet Desirability	API	Excipient
				mg	mg
real	T25E75-01	-	-	89.8	320.4
B1	T25E75-01	Sta/RB/UC/EC18/G1.7/KM(9)	1.000	88.6	316.2
B4	T25E75-01	Ref/PFF/UC/EC11/G2.2/KM(11)	1.000	89.5	322.6
D	T25E75-01	-	-	88.6	316.2
G	T25E75-01	-	-	88.6	316.2
real	T25E75-03	-	-	86.9	329.5
B1	T25E75-03	Sta/RB/UC/EC18/G1.7/KM(9)	0.982	90.0	329.0
B2	T25E75-03	Sta/RB/UC/G1.3/EC14/KM(7)	0.991	89.3	329.6
B3	T25E75-03	Sta/UC/UC/EC13/G2.7/KM(12)	0.958	91.1	327.6
B4	T25E75-03	Ref/PFF/UC/EC11/G2.2/KM(11)	0.946	91.5	327.5
C1	T25E75-03	Sta/RB/UC/EC17/G1.5/KM(12)	0.823	78.6	329.1
C2	T25E75-03	Sta/UC/UC/EC13/G2.5/KM(12)	0.904	93.0	326.2
API1	T25E75-03	Ref/PFF/UC/G3.3/EC13/KM(12)	0.985	84.1	332.5
API2	T25E75-03	Ref/PFF/UC/EC16/G3.7/KM(12)	0.789	96.3	322.4
T1	T25E75-03	Ref/RB/NLM/G1.9/EC18/KM(9)	1.000	86.2	326.6
T2	T25E75-03	Sta/RB/UC/EC18/G1.7/KM(10)	1.000	86.3	326.6
T3	T25E75-03	Ref/RB/NLM/G1.9/EC18/KM(11)	0.902	80.6	325.2
T4	T25E75-03	Ref/PFF/NLM/EC15/G2.7/KM(12)	0.801	96.1	323.8
T5	T25E75-03	Ref/PFF/UC/EC11/G2.3/KM(10)	0.700	98.4	322.0
T6	T25E75-03	Ref/UC/NLM/EC2/G2.3/MaE-iB//MaE	0.600	98.2	305.8
T7	T25E75-03	Ref/UC/NLM/ECequalize/G2.1/KM(11)	0.506	73.3	329.2
T8	T25E75-03	Ref/UC/NLM/EC2/G2.3/MaE//MiE	0.405	98.2	286.5
T9	T25E75-03	Ref/UC/NLM/EC2/G2.3/MaE//Y-iB	0.326	98.2	280.8
T10	T25E75-03	Ref/UC/NLM/ECequalize/G2.3/KM(6)	0.202	100.9	280.9
T11	T25E75-03	Ref/PFF/NLM/ECequalize/G2.1/KM(4)	0.127	73.0	275.5
D	T25E75-03	-	-	88.6	316.2
G	T25E75-03	-	-	88.6	316.2
real	T25E75-05	-	-	86.1	331.4
B1	T25E75-05	Sta/RB/UC/EC18/G1.7/KM(9)	0.955	90.4	330.3
B4	T25E75-05	Ref/PFF/UC/EC11/G2.2/KM(11)	0.928	91.4	329.4
D	T25E75-05	-	-	88.6	316.2
G	T25E75-05	-	-	88.6	316.2

**Table 2:** The selected parameters for the in silico calculations.

Parameter	Value
general	
cube-X, cube-Y, cube-Z	image size
unitcube	0.028 mm
iterations	43,200
timeformat	seconds
sampling interval	600
mixing parameter	0.05
theophylline	
identification number	1
density	1447.2 kg/m³
solubility	10.09 mg/mL
molar mass	180.16 Da
ethyl cellulose	
identification number	31
density	1139.9 kg/m³
solubility	0.00 mg/mL
molar mass	75,000.0 Da
contact angle	89°
porosity	calculated by Equation (2) (%)
pore size	0.5 μm
phosphate buffer	
identification number	200
density	1007.0 kg/m³
viscosity	0.0006865 Pa s
tension	67.9 mN/m
air	
identification number	0

**Table 3:** The calculated results of the comparison of the predicted dissolution profiles and the experimental one. The first row in a cell is the value of the Higuchi plot, the second row represents the value of the Korsmeyers–Peppas plot. ’NA’ indicates that a value was not calculated because either there were too few data points or it was not necessary, e.g., if the slopes were significantly different, the *p*-value of the axis intercept was not calculated.

Matrix	Tablet Desirability	$f_{1}$-Value	$f_{2}$-Value	Slope	Intercept	$R^{2}$	*p*-Value Slope	*p*-Value Intercept
real data	-	-	-	1.7869 0.4802	1.0926 0.3172	0.9996 0.9990	-	-
$T25E75-03_{API1}$	0.985	11.7	66.1	2.5605 0.6509	−10.7717 −0.0910	0.9993 0.9983	2.94 × 10${}^{-105}$ 2.14 × 10${}^{-75}$	NA NA
$T25E75-03_{API2}$	0.789	20.3	54.2	2.7863 0.6512	−11.0816 −0.0478	0.9992 0.9985	4.70 × 10${}^{-108}$ 1.06 × 10${}^{-74}$	NA NA
$T25E75-03_{C1}$	0.823	22.6	55.4	2.1661 0.7450	−15.2112 −0.4924	0.9959 0.9982	3.55 × 10${}^{-42}$ 2.51 × 10${}^{-82}$	NA NA
$T25E75-03_{C2}$	0.904	9.8	70.9	2.6728 0.7356	−16.739 −0.3548	0.9989 0.9997	9.86 × 10${}^{-100}$ 2.05 × 10${}^{-108}$	NA NA
$T25E75-03_{B1}$	0.982	10.3	69.4	2.5019 0.7382	−16.1765 −0.3962	0.9986 0.9997	6.51 × 10${}^{-86}$ 1.37 × 10${}^{-105}$	NA NA
$T25E75-03_{B2}$	0.991	10.6	68.9	2.4955 0.7384	−16.3043 −0.3996	0.9987 0.9997	9.48 × 10${}^{-86}$ 2.30 × 10${}^{-103}$	NA NA
$T25E75-03_{B3}$	0.958	9.5	71.5	2.6392 0.7369	−16.7893 −0.3667	0.9989 0.9997	4.32 × 10${}^{-97}$ 2.74 × 10${}^{-107}$	NA NA
$T25E75-03_{B4}$	0.946	17.0	57.9	2.7147 0.6526	−11.1982 −0.0675	0.9994 0.9984	6.12 × 10${}^{-112}$ 2.32 × 10${}^{-75}$	NA NA
$T25E75-03_{T1}$	1.000	12.5	63.9	2.7689 0.7000	−15.0743 −0.2217	0.9984 0.9995	1.85 × 10${}^{-96}$ 3.01 × 10${}^{-102}$	NA NA
$T25E75-03_{T2}$	1.000	14.0	64.3	2.3850 0.7458	−16.0278 −0.4445	0.9988 0.9992	9.05 × 10${}^{-80}$ 5.35 × 10${}^{-96}$	NA NA
$T25E75-03_{T3}$	0.902	8.7	72.2	2.5916 0.6875	−13.9408 −0.215	0.9967 0.9993	1.17 × 10${}^{-73}$ 4.16 × 10${}^{-95}$	NA NA
$T25E75-03_{T4}$	0.801	40.4	40.9	3.0837 0.6132	−8.3566 0.1323	0.9990 0.9989	2.12 × 10${}^{-102}$ 3.12 × 10${}^{-62}$	NA NA
$T25E75-03_{T5}$	0.700	21.8	52.8	2.8241 0.6520	−11.1975 −0.0436	0.9993 0.9985	3.96 × 10${}^{-110}$ 1.05 × 10${}^{-73}$	NA NA
$T25E75-03_{T6}$	0.600	30.7	45.6	3.0188 0.6516	−11.5268 −0.0083	0.9985 0.9995	1.03 × 10${}^{-96}$ 1.03 × 10${}^{-82}$	NA NA
$T25E75-03_{T7}$	0.506	7.7	75.5	2.5034 0.6785	−13.2446 −0.2039	0.9948 0.9981	1.44 × 10${}^{-59}$ 2.86 × 10${}^{-78}$	NA NA
$T25E75-03_{T8}$	0.405	48.7	36.8	3.3312 0.6346	−10.0969 0.1038	0.9996 0.9997	2.39 × 10${}^{-113}$ 8.02 × 10${}^{-73}$	NA NA
$T25E75-03_{T9}$	0.326	58.0	33.2	3.5779 0.6480	−11.2144 0.1001	0.9997 0.9997	2.04 × 10${}^{-112}$ 3.70 × 10${}^{-70}$	NA NA
$T25E75-03_{T10}$	0.202	57.7	33.3	3.5908 0.6508	−11.4484 0.0928	0.9996 0.9997	2.42 × 10${}^{-110}$ 1.00 × 10${}^{-70}$	NA NA
$T25E75-03_{T11}$	0.127	81.2	26.5	4.0747 0.6462	−11.1658 0.1777	0.9992 0.9998	3.76 × 10${}^{-100}$ 7.30 × 10${}^{-63}$	NA NA
$T25E75-03_{D}$	-	30.0	44.7	NA NA	NA NA	0.1855 0.3603	4.12 × 10${}^{-81}$ 1.72 × 10${}^{-93}$	NA NA
$T25E75-03_{D2}$	-	125.4	18.4	3.1517 0.2779	17.4812 1.1372	0.9975 0.9913	1.08 × 10${}^{-65}$ 4.85 × 10${}^{-56}$	NA NA
$T25E75-03_{G}$	-	111.3	21.2	5.9146 0.3874	11.9812 1.0768	0.9559 0.9687	2.15 × 10${}^{-38}$ 5.38 × 10${}^{-14}$	NA NA
$T25E75-03_{G2}$	-	146.5	15.3	5.2757 0.3997	9.7346 0.9971	0.9793 0.9841	1.16 × 10${}^{-47}$ 5.95 × 10${}^{-16}$	NA NA

## Data Availability

The virtual matrices and the XμCT images of the nine presented tablets inclusive raw data of the XμCT measurement will be provided in a repository due to storage capacity issues. The link to the repository is available on request.

## Appendix A. Tables

**Table A1:** The selected images for the in silico calculations. The line called real displays the determined real data of the tablet.

Name	Tablet	Pathway	Tablet Desirability	API	Excipient
				mg	mg
real	T50E50-01	-	-	203.8	229.7
B1	T50E50-01	Ref/UC/NLM/EC2/G2.3/H-iW//O-iB	0.995	201.4	235.3
B2	T50E50-01	Ref/PFF/UC/G2.2/EC11/KM(11)	1.000	207.2	229.9
D	T50E50-01	-	-	88.6	316.2
G	T50E50-01	-	-	88.6	316.2
real	T50E50-03	-	-	199.9	212.4
B1	T50E50-03	Ref/UC/NLM/EC2/G2.3/H-iW//O-iB	1.000	196.9	215.9
B2	T50E50-03	Ref/PFF/UC/G2.2/EC11/KM(11)	0.990	194.3	209.5
B3	T50E50-03	Ref/PFF/UC/G3.7/EC16/H-iW//Y-iBiW	0.978	194.2	217.4
B4	T50E50-03	Ref/UC/NLM/G3.0/EC16/ID-iB//O-iB	0.989	195.7	217.8
C1	T50E50-03	Sta/RB/UC/EC17/G1.5/KM(12)	0.747	218.3	198.9
C2	T50E50-03	Sta/UC/UC/EC13/G2.5/KM(12)	0.683	175.0	202.4
API1	T50E50-03	Ref/PFF/UC/G3.3/EC13/KM(12)	0.987	205.2	207.9
API2	T50E50-03	Ref/PFF/UC/EC16/G3.7/KM(12)	0.962	206.7	206.3
T1	T50E50-03	Ref/UC/NLM/EC15/G2.9/H-iBiW//ID-iB	1.000	200.0	212.4
T2	T50E50-03	Sta/PFF/NLM/EC15/G2.8/KM(11)	1.000	200.9	213.4
T3	T50E50-03	Ref/PFF/NLM/EC18/G1.6/O-iB//Y-iBiW	0.900	209.5	202.7
T4	T50E50-03	Ref/PFF/UC/G3.5/EC19/KM(5)	0.800	213.1	197.7
T5	T50E50-03	Sta/RB/UC/G1.7/EC18/MaE-iW//MiE-iB	0.700	181.6	228.2
T6	T50E50-03	Ref/PFF/NLM/EC17/G3/MaE-iB//MiE-iB	0.600	219.1	188.4
T7	T50E50-03	Ref/UC/NLM/G3.1/EC18/MaE-iBiW//MiE-iB	0.500	218.8	182.8
T8	T50E50-03	Sta/RB/UC/G1.6/EC17/D-iB//MaE-iBiW	0.400	230.0	187.4
T9	T50E50-03	Ref/PFF/NLM/EC18/G3.1/MiE-iB//O	0.300	218.9	177.8
T10	T50E50-03	Ref/UC/NLM/EC16/G3.0/D//MaE	0.201	232.0	179.7
T11	T50E50-03	Ref/UC/NLM/G3.2/EC18/D//MaE	0.101	232.8	178.2
D	T50E50-03	-	-	88.6	316.2
G	T50E50-03	-	-	88.6	316.2
real	T50E50-05	-	-	201.2	218.3
B1	T50E50-05	Ref/UC/NLM/EC2/G2.3/H-iW//O-iB	1.000	204.3	217.7
B2	T50E50-05	Ref/PFF/UC/G2.2/EC11/KM(11)	1.000	199.8	220.6
D	T50E50-05	-	-	88.6	316.2
G	T50E50-05	-	-	88.6	316.2

**Table A2:** The selected images for the in silico calculations. The line called real displays the determined real data of the tablet.

Name	Tablet	Pathway	Tablet Desirability	API	Excipient
				mg	mg
real	T75E25-01	-	-	319.6	109.1
B1	T75E25-01	Ref/PFF/NLM/G1.5/EC16/KM(12)	1.000	313.6	108.7
B4	T75E25-01	Sta/RB/UC/EC18/G1.7/KM(9)	0.986	314.1	112.2
D	T75E25-01	-	-	88.6	316.2
G	T75E25-01	-	-	88.6	316.2
real	T75E25-03	-	-	318.3	115.9
B1	T75E25-03	Ref/PFF/NLM/G1.5/EC16/KM(12)	0.998	317.1	118.3
B2	T75E25-03	Ref/PFF/NLM/G2.7/EC15/KM(12)	1.000	320.5	116.9
B3	T75E25-03	Ref/PFF/UC/G2.0/EC13/KM(12)	1.000	320.5	117.4
B4	T75E25-03	Sta/RB/UC/EC18/G1.7/KM(9)	1.000	316.5	115.8
C1	T75E25-03	Sta/RB/UC/EC17/G1.5/KM(12)	0.928	308.4	121.0
C2	T75E25-03	Sta/UC/UC/EC13/G2.5/KM(12)	0.975	310.9	119.4
API1	T75E25-03	Ref/PFF/UC/G3.3/EC13/KM(12)	1.000	321.7	115.0
API2	T75E25-03	Ref/PFF/UC/EC16/G3.7/KM(12)	1.000	321.6	114.6
T1	T75E25-03	Ref/RB/NLM/G3.0/EC17/KM(12)	1.000	320.6	116.7
T2	T75E25-03	Ref/UC/NLM/EC16/G3.0/H-iB//MiE-iB	1.000	319.9	116.4
T3	T75E25-03	Sta/UC/UC/G2.7/EC13/P//T	0.900	316.7	124.2
T4	T75E25-03	Sta/RB/UC/G3.2/EC17/H-iW//R-iBiW	0.800	308.6	126.9
T5	T75E25-03	Ref/PFF/NLM/G1.6/EC17/H-iBiW//M	0.700	281.7	112.2
T6	T75E25-03	Sta/PFF/UC/G3.0/EC17/D-iW//O-iBiW	0.600	285.9	126.7
T7	T75E25-03	Ref/PFF/NLM/EC18/G3.1/L-iB//P-iW	0.500	345.3	99.5
T8	T75E25-03	Sta/UC/UC/G3.0/EC17/L//Y-iB	0.400	348.9	98.1
T9	T75E25-03	Sta/UC/UC/EC16/G2.8/M-iBiW//M-iW	0.301	275.4	97.1
T10	T75E25-03	Sta/UC/UC/EC16/G2.8/M-iW//R-iW	0.201	267.0	97.9
T11	T75E25-03	Ref/PFF/NLM/EC18/G1.7/M-iW//P-iBiW	0.102	266.0	133.7
D	T75E25-03	-	-	88.6	316.2
G	T75E25-03	-	-	88.6	316.2
real	T75E25-05	-	-	86.1	331.4
B1	T75E25-05	Ref/PFF/NLM/G1.5/EC16/KM(12)	1.000	307.3	116.3
B4	T75E25-05	Sta/RB/UC/EC18/G1.7/KM(9)	1.000	311.1	115.1
D	T75E25-05	-	-	88.6	316.2
G	T75E25-05	-	-	88.6	316.2

**Table A3:** The calculated results of the comparison of the predicted dissolution profiles and the experimental one. The first row in a cell is the value of the Higuchi plot, the second row represents the value of the Korsmeyers–Peppas plot. ’NA’ indicates that a value was not calculated because either there were too few data points or it was not necessary, e.g., if the slopes were significantly different, the *p*-value of the axis intercept was not calculated.

${\mathrm{Batch}}_{\mathrm{Matrix}}$	$f_{1}$-Value	$f_{2}$-Value	Slope	Intercept	$R^{2}$	*p*-Value Slope	*p*-Value Intercept
$T25E75_{real}$	-	-	1.7517 0.4781	1.1117 0.3143	0.9993 0.9985	-	-
$T25E75_{B1}$	10.6	69.6	2.5137 0.7463	−17.0895 −0.4257	0.9964 0.9994	2.76 × 10${}^{-68}$ 6.19 × 10${}^{-94}$	NA NA
$T25E75_{B4}$	17.3	58.0	2.6400 0.6396	−10.3343 −0.0398	0.9996 0.9991	3.98 × 10${}^{-116}$ 2.05 × 10${}^{-77}$	NA NA
$T25E75_{D}$	33.3	42.9	NA NA	NA NA	NA NA	NA NA	NA NA
$T25E75_{G}$	122.5	19.5	6.8476 0.4314	8.0960 1.0233	0.9621 0.9703	1.55 × 10${}^{-41}$ 8.99 × 10${}^{-5}$	NA NA
$T50E50_{real}$	-	-	2.2279 0.4709	2.3324 0.4465	0.9996 0.9997	-	-
$T50E50_{B1}$	54.5	29.3	4.8015 0.7592	−19.5489 −0.0649	0.9993 0.9995	2.77 × 10${}^{-89}$ 1.12 × 10${}^{-83}$	NA NA
$T50E50_{B2}$	55.3	29.4	4.7346 0.7169	−16.0672 0.0573	0.9996 0.9993	5.57 × 10${}^{-91}$ 8.84 × 10${}^{-76}$	NA NA
$T50E50_{D}$	125.6	13.1	NA NA	NA NA	NA NA	NA NA	NA NA
$T50E50_{G}$	128.1	12.8	NA NA	NA NA	NA NA	NA NA	NA NA
$T75E25_{real}$	-	-	5.6369 0.7242	−16.7140 0.1437	0.9955 0.9992	-	-
$T75E25_{B1}$	7.6	62.9	5.6819 0.7819	−21.3730 −0.0204	0.9999 0.9983	6.73 × 10${}^{-1}$ 4.86 × 10${}^{-6}$	3.65 × 10${}^{-4}$ NA
$T75E25_{B4}$	7.7	62.1	5.6879 0.7980	−22.5207 −0.0669	0.9998 0.9985	6.18 × 10${}^{-1}$ 3.19 × 10${}^{-8}$	1.33 × 10${}^{-5}$ NA
$T75E25_{D}$	42.5	20.8	NA NA	NA NA	NA NA	NA NA	NA NA
$T75E25_{G}$	42.4	20.9	NA NA	NA NA	NA NA	NA NA	NA NA

**Table A4:** The calculated results of the comparison of the predicted dissolution profiles and the experimental one. The first row in a cell is the value of the Higuchi plot, the second row represents the value of the Korsmeyers–Peppas plot. ’NA’ indicates that a value was not calculated because either there were too few data points or it was not necessary, e.g., if the slopes were significantly different, the *p*-value of the axis intercept was not calculated.

Matrix	Tablet Desirability	$f_{1}$-Value	$f_{2}$-Value	Slope	Intercept	$R^{2}$	*p*-Value Slope	*p*-Value Intercept
real data	-	-	-	2.1984 0.4677	2.4137 0.4501	0.9998 0.9996	-	-
$T50E50-03_{API1}$	0.987	59.9	28.0	4.9139 0.7326	−17.3007 0.0331	0.9996 0.9993	3.33 × 10${}^{-98}$ 4.62 × 10${}^{-74}$	NA NA
$T50E50-03_{API2}$	0.962	60.3	27.9	4.9299 0.7324	−17.3387 0.0351	0.9996 0.9993	9.79 × 10${}^{-99}$ 1.01 × 10${}^{-73}$	NA NA
$T50E50-03_{C1}$	0.747	61.3	27.1	5.0006 0.7760	−20.7285 −0.0873	0.9993 0.9995	9.77 × 10${}^{-95}$ 2.35 × 10${}^{-79}$	NA NA
$T50E50-03_{C2}$	0.683	62.9	26.6	5.0293 0.7707	−19.7255 −0.0614	0.9989 0.9996	1.19 × 10${}^{-91}$ 1.72 × 10${}^{-81}$	NA NA
$T50E50-03_{B1}$	1.000	57.6	28.3	4.9234 0.7770	−20.7390 −0.1004	0.9990 0.9996	5.67 × 10${}^{-93}$ 2.35 × 10${}^{-82}$	NA NA
$T50E50-03_{B2}$	0.990	59.7	28.0	4.9072 0.7333	−17.3619 0.0299	0.9995 0.9995	1.19 × 10${}^{-96}$ 8.14 × 10${}^{-76}$	NA NA
$T50E50-03_{B3}$	0.978	57.3	28.9	4.8021 0.7280	−16.9167 0.0325	0.9995 0.9994	8.54 × 10${}^{-98}$ 2.46 × 10${}^{-76}$	NA NA
$T50E50-03_{B4}$	0.989	57.0	28.5	4.9549 0.7912	−21.7382 −0.1390	0.9990 0.9995	4.46 × 10${}^{-95}$ 5.69 × 10${}^{-84}$	NA NA
$T50E50-03_{T1}$	1.000	58.7	28.0	4.9945 0.7849	−21.3789 −0.1153	0.9991 0.9995	1.27 × 10${}^{-94}$ 4.88 × 10${}^{-82}$	NA NA
$T50E50-03_{T2}$	1.000	60.4	27.9	4.9825 0.7323	−17.4912 0.0402	0.9996 0.9992	7.95 × 10${}^{-100}$ 8.65 × 10${}^{-73}$	NA NA
$T50E50-03_{T3}$	0.900	62.8	27.1	5.0622 0.7325	−17.5489 0.0495	0.9997 0.9991	7.37 × 10${}^{-99}$ 8.86 × 10${}^{-71}$	NA NA
$T50E50-03_{T4}$	0.800	62.9	27.0	5.0410 0.7373	−17.7432 0.0345	0.9996 0.9992	6.41 × 10${}^{-98}$ 5.29 × 10${}^{-72}$	NA NA
$T50E50-03_{T5}$	0.700	51.5	30.4	4.6601 0.7755	−20.3361 −0.1303	0.9983 0.9998	2.40 × 10${}^{-88}$ 1.31 × 10${}^{-90}$	NA NA
$T50E50-03_{T6}$	0.600	67.1	25.8	5.2604 0.7395	−18.3228 0.0508	0.9998 0.9989	6.92 × 10${}^{-100}$ 1.91 × 10${}^{-68}$	NA NA
$T50E50-03_{T7}$	0.500	68.9	24.9	5.3317 0.7872	−21.0379 −0.0717	0.9994 0.9992	9.86 × 10${}^{-95}$ 4.53 × 10${}^{-75}$	NA NA
$T50E50-03_{T8}$	0.400	64.6	26.0	5.0739 0.7787	−20.2307 −0.0785	0.9991 0.9995	3.18 × 10${}^{-93}$ 5.39 × 10${}^{-81}$	NA NA
$T50E50-03_{T9}$	0.300	72.5	24.1	5.4448 0.7569	−18.6035 0.0331	0.9996 0.9990	1.09 × 10${}^{-96}$ 2.74 × 10${}^{-70}$	NA NA
$T50E50-03_{T10}$	0.201	68.0	25.1	5.3058 0.7854	−21.1099 −0.0725	0.9994 0.9992	1.25 × 10${}^{-96}$ 2.56 × 10${}^{-76}$	NA NA
$T50E50-03_{T11}$	0.101	68.5	25.0	5.3330 0.7857	−21.2127 −0.0709	0.9994 0.9991	1.40 × 10${}^{-97}$ 9.34 × 10${}^{-76}$	NA NA
$T50E50-03_{D}$	-	129.5	12.7	NA NA	NA NA	NA NA	NA NA	NA NA
$T50E50-03_{G}$	-	131.3	12.4	NA NA	NA NA	NA NA	NA NA	NA NA

**Table A5:** The calculated results of the comparison of the predicted dissolution profiles and the experimental one. The first row in a cell is the value of the Higuchi plot, the second row represents the value of the Korsmeyers–Peppas plot. ’NA’ indicates that a value was not calculated because either there were too few data points or it was not necessary, e.g., if the slopes were significantly different, the *p*-value of the axis intercept was not calculated.

Matrix	Tablet Desirability	$f_{1}$-Value	$f_{2}$-Value	Slope	Intercept	$R^{2}$	*p*-Value Slope	*p*-Value Intercept
$T75E25-03_{API1}$	1.000	5.8	68.8	5.7043 0.7971	−22.1516 −0.0583	0.9999 0.9980	7.64 × 10${}^{-1}$ 1.38 × 10${}^{-5}$	2.92 × 10${}^{-4}$ NA
$T75E25-03_{API2}$	1.000	5.7	69.1	5.7087 0.7966	−22.1438 −0.0567	0.9999 0.9980	7.20 × 10${}^{-1}$ 1.61 × 10${}^{-5}$	2.97 × 10${}^{-4}$ NA
$T75E25-03_{C1}$	0.928	6.5	66.3	5.6680 0.8111	−23.0211 −0.1025	0.9998 0.9983	8.53 × 10${}^{-1}$ 1.30 × 10${}^{-7}$	1.22 × 10${}^{-5}$ NA
$T75E25-03_{C2}$	0.975	6.3	67.0	5.6823 0.8098	−23.0180 −0.0980	0.9998 0.9983	9.93 × 10${}^{-1}$ 1.85 × 10${}^{-7}$	1.20 × 10${}^{-5}$ NA
$T75E25-03_{B1}$	0.998	6.5	66.8	5.6632 0.7917	−21.9957 −0.0508	0.9999 0.9978	7.98 × 10${}^{-1}$ 1.08 × 10${}^{-4}$	2.90 × 10${}^{-4}$ NA
$T75E25-03_{B2}$	1.000	6.3	67.3	5.6875 0.7991	−22.1820 −0.0649	0.9999 0.9980	9.38 × 10${}^{-1}$ 8.23 × 10${}^{-6}$	2.75 × 10${}^{-4}$ NA
$T75E25-03_{B3}$	1.000	6.1	67.8	5.6941 0.7996	−22.2315 −0.0656	0.9999 0.9980	8.69 × 10${}^{-1}$ 7.00 × 10${}^{-6}$	2.28 × 10${}^{-4}$ NA
$T75E25-03_{B4}$	1.000	6.1	67.4	5.7011 0.8119	−23.1813 −0.1020	0.9998 0.9982	7.91 × 10${}^{-1}$ 1.52 × 10${}^{-7}$	6.73 × 10${}^{-6}$ NA
$T75E25-03_{T1}$	1.000	6.6	65.9	5.6849 0.8141	−23.1965 −0.1087	0.9998 0.9981	9.65 × 10${}^{-1}$ 1.55 × 10${}^{-7}$	6.33 × 10${}^{-6}$ NA
$T75E25-03_{T2}$	1.000	6.6	66.1	5.6917 0.8138	−23.2188 −0.1075	0.9998 0.9982	8.91 × 10${}^{-1}$ 1.31 × 10${}^{-7}$	6.01 × 10${}^{-6}$ NA
$T75E25-03_{T3}$	0.900	9.5	58.2	6.0098 0.9190	−30.3709 −0.3713	0.9998 0.9971	1.49 × 10${}^{-4}$ 1.37 × 10${}^{-13}$	NA NA
$T75E25-03_{T4}$	0.800	7.2	64.3	5.6312 0.8146	−23.0534 −0.1147	0.9997 0.9984	5.01 × 10${}^{-1}$ 2.82 × 10${}^{-8}$	1.34 × 10${}^{-5}$ NA
$T75E25-03_{T5}$	0.700	1.8	86.8	6.0766 0.8091	−22.5060 −0.0438	0.9998 0.9979	3.23 × 10${}^{-5}$ 2.31 × 10${}^{-6}$	NA NA
$T75E25-03_{T6}$	0.600	3.1	79.8	5.7914 0.7993	−21.3596 −0.0444	0.9998 0.9980	1.57 × 10${}^{-1}$ 1.39 × 10${}^{-5}$	3.17 × 10${}^{-3}$ NA
$T75E25-03_{T7}$	0.500	6.0	68.2	5.8670 0.8364	−24.5041 −0.1474	0.9999 0.9971	1.92 × 10${}^{-2}$ 5.34 × 10${}^{-8}$	1.45 × 10${}^{-7}$ NA
$T75E25-03_{T8}$	0.400	6.7	65.1	6.0149 0.8676	−27.8541 −0.2317	0.9999 0.9972	1.42 × 10${}^{-4}$ 1.38 × 10${}^{-10}$	NA NA
$T75E25-03_{T9}$	0.301	5.3	70.5	6.4250 0.8283	−24.3255 −0.0636	0.9998 0.9979	3.57 × 10${}^{-9}$ 4.19 × 10${}^{-8}$	NA NA
$T75E25-03_{T10}$	0.201	6.2	67.1	6.5041 0.8330	−24.4633 −0.0649	0.9998 0.9981	3.22 × 10${}^{-9}$ 6.95 × 10${}^{-9}$	NA NA
$T75E25-03_{T11}$	0.102	3.0	81.0	5.7810 0.7968	−21.2583 −0.0392	0.9997 0.9983	2.07 × 10${}^{-1}$ 8.44 × 10${}^{-6}$	5.01 × 10${}^{-3}$ NA
$T75E25-03_{D}$	-	46.0	20.3	NA NA	NA NA	NA NA	NA NA	NA NA
$T75E25-03_{G}$	-	45.8	20.4	NA NA	NA NA	NA NA	NA NA	NA NA
